# Activation of spleen cells by ArtinM may account for its immunomodulatory properties

**DOI:** 10.1007/s00441-014-1879-8

**Published:** 2014-05-20

**Authors:** Thiago Aparecido da Silva, Maria Aparecida de Souza, Nerry Tatiana Cecílio, Maria Cristina Roque-Barreira

**Affiliations:** Departamento de Biologia Celular e Molecular e Bioagentes Patogênicos, Faculdade de Medicina de Ribeirão Preto, Universidade de São Paulo, Avenida Bandeirantes 3900, 14049-900 Ribeirão Preto, SP Brazil

**Keywords:** ArtinM, Spleen cells, T lymphocytes, Carbohydrate recognition, Immunomodulation

## Abstract

ArtinM is a D-mannose-binding lectin extracted from *Artocarpus heterophyllus* that promotes interleukin-12 production by macrophages and dendritic cells. This property is considered responsible for T helper 1 immunity induced in vivo after ArtinM administration. In this study, we investigated the effect of native (jArtinM) and recombinant (rArtinM) forms of lectin on murine spleen cells and isolated T lymphocytes. We found that ArtinM binds to the surface of spleen cells. This interaction, which was blocked by D-mannose, induced cell activation, as manifested by increased mitochondrial activity, interleukin-2 production, and cell proliferation. We verified that a 30-times higher concentration of rArtinM was required to trigger optimal activation of spleen cells compared with that needed with jArtinM, although these proteins have identical sugar recognition properties and use the same signaling molecules to trigger cell activation. Because the distinction between native and recombinant is restricted to their tertiary structure (tetrameric and monomeric, respectively), we postulated that the multi-valence of jArtinM accounts for its superiority in promoting clustering of cell surface glycoreceptors and activation. The jArtinM and rArtinM activation effect exerted on spleen cells was reproduced on purified CD4^+^ T cells. Our results suggest that ArtinM interaction with T cells leads to responses that may act in concert with the interleukin-12 produced by antigen-presenting cells to modulate immunity toward the T helper 1 axis. Further studies are necessary to dissect ArtinM/T-cell interactions to more fully understand the immunomodulation induced by carbohydrate recognition.

## Introduction

Lectins are proteins with at least one non-catalytic domain that binds reversibly and specifically to a mono- or oligosaccharide (Peumans and Van Damme 1995). They are ubiquitous in nature, being found in organisms from viruses to humans (Sharon 2008). Lectins are involved in numerous biological activities, including promotion of cell–cell and cell–extracellular matrix interactions, complement system activation, induction of leukocyte activation and migration, induction of cytokine production, adhesion of pathogens to host cells, and induction of cell death (Sharon and Lis 2004). Most often, the biological activities of lectins depend on their carbohydrate recognition domain (CRD), which interacts with cell surface glycoproteins (Baba et al. 2007) that trigger cell signaling.

ArtinM, a D-mannose-binding lectin, obtained from the seeds of *Artocarpus heterophyllus*, is organized as a homotetramer formed by 16-kDa non-glycosylated subunits (Santos-de-Oliveira et al. 1994). Each polypeptide chain contains a CRD with affinity to Manα 1–3 [Manα 1–6] Man, which corresponds to the core N-linked oligosaccharides. The following activities are attributed to ArtinM: (1) neutrophil activation, triggered by binding to N-glycans on Toll-like receptor 2 (TLR2) and chemokine receptor (CXCR2) (Ganiko et al. 1998; Pereira-da-Silva et al. 2006; Santos-de-Oliveira et al. 1994), (2) mast cell degranulation induced by interactions with *N*-glycans of FcεR or N-glycans of immunoglobulin E-linked FcεR (Moreno et al. 2003), and (3) production of interleukin (IL)-12 stimulated by recognition of *N*-linked glycans of the TLR2 ectodomain on the surface of macrophages and dendritic cells (Coltri et al. 2008). The induction of IL-12 production is considered a central effect because ArtinM administration drives immunity toward the T helper (Th) 1 axis (Panunto-Castelo et al. 2001; Teixeira et al. 2006), conferring resistance to infections by intracellular pathogens such as *Leishmania major* (Panunto-Castelo et al. 2001), *L. amazonensis* (Teixeira et al. 2006), *Paracoccidioides brasiliensis* (Coltri et al. 2008, 2010), *Neospora caninum* (Cardoso et al. 2011), and *Candida albicans* (Custodio et al. 2011).

The ArtinM immunomodulatory property is exerted by both lectin forms, native (jArtinM) and recombinant (rArtinM) (daSilva et al. 2005; Pranchevicius et al. 2012), which structurally differ in terms of oligomerization. In opposition to the tetrameric structure of native ArtinM, the recombinant counterpart, obtained by expression in *Escherichia coli*, is monomeric. The availability of native and recombinant ArtinM provides convenient tools with which to investigate the requirements for triggering cell signaling.

Regarding the mechanisms accounting for ArtinM immunomodulatory properties, IL-12 production due to lectin interaction with TLR2 on macrophages and dendritic cells has been well exploited. However, the possibility that the effects of ArtinM extend to lymphoid cells and establish interactions that contribute to immunomodulation has not been investigated. In the present study, the activities exerted by native and recombinant ArtinM on murine spleen cells were examined. A direct effect on T cells was verified, providing new elements for full comprehension of the immunomodulation promoted by ArtinM and its potential application to the design of immunotherapeutic strategies.

## Materials and methods

### Ethics statement

Animal studies were approved by the Ethical Committee of Ethics in Animal Research of the College of Medicine of Ribeirão Preto of the University of São Paulo and were conducted in accordance with the Ethical Principles in Animal Research adopted by the Brazilian College of Animal Experimentation, Protocol no. 082/2012.

### Animals

Male BALB/c, C57BL/6, TLR2 KO, and TLR4 KO mice were acquired from the animal house of the Campus of Ribeirão Preto, University of São Paulo, Ribeirão Preto, São Paulo, Brazil, and housed in the animal facility of the Molecular and Cellular Biology Department of the Faculty of Medicine of Ribeirão Preto, University of São Paulo, under optimized hygienic conditions. All experiments were conducted in accordance with the ethical guidelines of the animal studies ethics committee. The experimental mice were used at 6–8 weeks of age.

### Lectins

jArtinM was purified as previously described (Santos-de-Oliveira et al. 1994) from the saline extract of *A. heterophyllus* (jackfruit) seeds via affinity chromatography on sugar columns. rArtinM was expressed in *E. coli* BL21 and purified as previously reported (daSilva et al. 2005). Before use, preparations of jArtinM and rArtinM were incubated for 1 h with polymyxin solution (Sigma-Aldrich, St. Louis, MO, USA). Concanavalin A (ConA) from *Canavalia ensiformis* was purchased from Sigma Chemical.

### Suspensions of spleen cells and isolated CD4^+^ T cells

Mice spleens were removed aseptically and transferred to a Petri dish where they were soaked and filtered in a 40-μm nylon cell strainer (BD Biosciences, San Diego, CA, USA) containing Roswell Park Memorial Institute (RPMI) 1640 medium. The cellular suspension was centrifuged at 300*g* (10 min at 4 °C) to yield a pellet. The suspension was erythrocyte-depleted with lysing buffer (9 parts 0.16 M ammonium chloride and one part 0.17 M Tris–HCl, pH 7.5) for 10 min at 4 °C. The spleen cells were then washed twice in 10 % fetal cow serum (FCS)/RPMI 1640 and centrifuged at 300*g* (10 min at 4 °C). Cells were counted in a Neubauer chamber, and their viability was determined using the trypan blue exclusion method. Viability of the spleen cells was greater than 90 %.

CD4^+^ T cells were isolated from spleen cell suspensions using CD4^+^ T cell isolation kits II and MS columns, both from Miltenyi-Biotec (Auburn, CA, USA) according to the manufacturer’s instructions. To assess purity, negatively selected cells were stained with anti-CD4 PE-Cy5 antibody (BD Biosciences) and analyzed with flow cytometry (Guava easyCyte, Guava Technologies, Millipore). Purity grades of 92–95 % were achieved.

### IL-2 measurement in cell supernatants

Spleen cells (1.5 × 10^6^/mL) were cultured in the presence of jArtinM (0.14–156.00 nM), rArtinM (0.56–625.00 nM) or ConA (49.0 nM) in 96-well microplates. After 12, 24, 48, and 72 h of incubation, the spleen cells were centrifuged (300*g*, 10 min at room temperature), and the supernatants were collected to measure IL-2 levels using an enzyme-linked immunosorbent assay from Kit OptEIA (BD Biosciences) according to the manufacturer’s instructions. Similar procedures were performed to determine IL-2 levels in the supernatants of isolated CD4^+^ T cells.

### 3-(4,5-dimethyl-thiazol-2-yl)-2,5-diphenyltetrazolium bromide (MTT) assay

Spleen cells distributed and stimulated as described for IL-2 detection were assayed for the reduction of MTT (Sigma-Aldrich) and production of formazan crystals (Mosmann 1983). MTT solution (500 μg/mL in RPMI 1640 containing 10 % FCS) was added (100 μL) to the wells containing spleen cells. The microplates were incubated for 3 h 30 min at 37 °C in a humidified atmosphere of 5 % CO_2_. The cells were then centrifuged (300*g*, 10 min at room temperature), the supernatants were removed, and 100 μL dimethyl sulfoxide was added to the wells. After overnight incubation at room temperature, the absorbance was read at 570 nm using a spectrophotometer (Power Wave X; BioteK Instruments). Mitochondrial activity was expressed as absorbance variation (as a percentage) in relation to the negative control and calculated as follows: [(optical density value in stimulated cells/average optical density value in unstimulated cells) −1] × 100. The positive or negative percentage values represent the increase or decrease in mitochondrial activity related to unstimulated cells.

### Cell proliferation assay

Spleen cells (1.5 × 10^6^/mL) were distributed in a 96-well microplate and cultured in the presence of jArtinM (2.25 nM or 4.50 nM), rArtinM (78.00 nM or 156.00 nM), or ConA (24.5 nM). The cells were cultured for 48 h, and, during the last 12 h of incubation, tritiated thymidine ([3H]-TdR; Amersham Bioscience, Boston, MA, USA) was added to the wells (0.5 μCi/well). Cell proliferation was assessed by measuring [3H]-TdR incorporation, and the results were expressed in counts per minute (cpm).

### Sugar inhibition assay of ArtinM activities

D-mannose (100 mM) or D-galactose (100 mM) was added to jArtinM (2.25 nM), rArtinM (78.00 nM), or phorbol myristate acetate (PMA; 81.0 nM) plus ionomycin (1.0 μM). After 30 min of incubation at 4 °C, the mixtures were distributed in the wells of a microplate containing spleen cell suspension (1.5 × 10^6^ cell/mL) in a final concentration of 50 mM sugars. The microplates were incubated at 37 °C in a humidified atmosphere of 5 % CO_2_. The cells and supernatants were analyzed for mitochondrial activity and IL-2 levels following the protocols described earlier.

### Cell activation after treatment with inhibitors of cell signaling molecules

Spleen cells (1.5 × 10^6^/mL) were incubated with the protein tyrosine kinase inhibitor genistein (20 μg/mL), the p42/44 mitogen-associated protein kinase (MAPK) inhibitor PD98059 (20 μM), the p38 MAPK inhibitor SB202190 (20 μM), the protein kinase C inhibitor H-7 (20 μM), or the c-Jun N-terminal kinase (JNK) inhibitor SP600125 (25 μM) for 3 h 30 min in RPMI 1640 with 10 % FCS at 37 °C in a humidified atmosphere of 5 % CO_2_. The cells were then stimulated with jArtinM (2.25 nM), rArtinM (78.00 nM), PMA (81.0 nM) plus ionomycin (1.0 μM), or ConA (49.0 nM) for 48 h. The cells and supernatants were analyzed for mitochondrial activity and IL-2 levels following protocols described earlier.

### Flow cytometry analysis of ArtinM binding on spleen cells

Biotinylated ArtinM (20 μg/mL) was incubated for 45 min with D-mannose or D-galactose (1 mM, 10 mM, 20 mM, 30 mM, or 50 mM) or with medium alone. These mixtures were added to fixed (3 % formaldehyde-phosphate-buffered saline) spleen cells (1.5 × 10^6^/mL) that were then incubated for 30 min at room temperature. After two washes with phosphate-buffered saline, bound biotinylated ArtinM on spleen cells was reacted with streptavidin-fluorescein isothiocyanate (strp/FITC; 5 μg/mL; Invitrogen) for 40 min and fluorescence staining was analyzed with flow cytometry (Guava EasyCyte). The percentage of stained spleen cells was then determined. To evaluate ArtinM binding on T cells, we treated the spleen cells with bound ArtinM for an additional 40 min with anti-CD3 PE-Cy5 antibody (4 μg/mL; BD Biosciences) and performed flow cytometry analysis. The percentage of double-positive cells for each experimental condition was then analyzed.

### Competition assays between an anti-CD3 antibody and ArtinM in relation to CD4^+^ T cells

The binding of ArtinM to CD3 receptor was determined on CD4^+^ T cells isolated from spleen cell suspensions using CD4^+^ T cell isolation kits II and MS columns from Miltenyi-Biotec according to the manufacturer’s instructions. In the first assay, the cells were fixed and incubated with ArtinM (25 μg/mL) for 40 min at 4 °C. After two washes with phosphate-buffered saline (PBS), the cells were incubated with an anti-CD3 PE-Cy5 antibody (10 μg/mL; clone 17A2; BD Biosciences) for 40 min at 4 °C. The percentage of labeled cells was determined using flow cytometry (Guava EasyCyte). A functional inhibition assay was carried out by pre-incubating CD4^+^ T cells with either the anti-CD3 antibody (5 μg/mL) or an anti-CD28 antibody (5 μg/mL; clone 37.51; BD Biosciences) for 40 min at 4 °C. After washing with RPMI medium, the cells were stimulated for 48 h with jArtinM (78 nM). The culture supernatants were tested by means of ELISA for IL-2.

### Statistical analysis

Results are presented as means ± standard error of the mean and all data were analyzed using Prism (Graph Pad Software). Statistical determinations of the difference between means of groups were performed with analysis of variance (1-way) followed by Bonferroni’s multiple comparison test. Differences that provided *p* < 0.05 were considered statistically significant.

## Results

### ArtinM binding on murine spleen cells

**Fig. 1:**
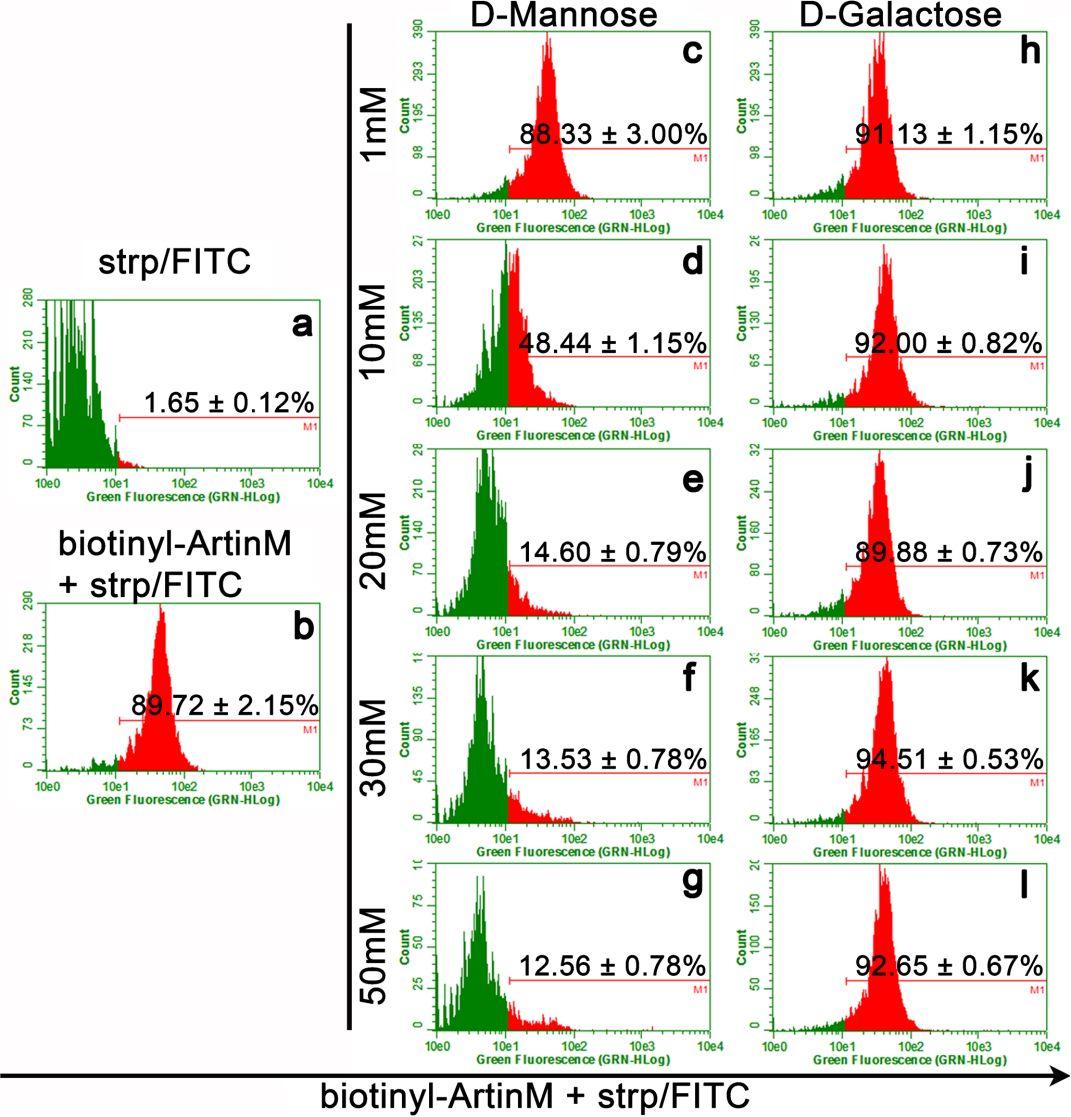
ArtinM binds to the surface of spleen cells through carbohydrate recognition. Biotinylated jArtinM (20 μg/mL) was incubated with the indicated concentrations of D-mannose (**c**–**g**) or D-galactose (**h**–**l**). The resulting mixtures were added to a suspension (1.5 × 10^6^ cells/mL) of fixed spleen cells, which were obtained from BALB/c mice. ArtinM binding was revealed by reaction with streptavidin-FITC (5 μg/mL). Streptavidin-FITC alone was used as a negative control (**a**). The cells were washed and analyzed for fluorescence intensity by flow cytometry. Figures show the percentage of positive cells for ArtinM binding for each experimental condition (**b**–**l**). The results are expressed as means ± SEM and represent 3 independent experiments

The binding of ArtinM on murine spleen cells was evaluated by incubating the cells with biotinylated ArtinM (20 μg/mL) and reacting them with strp/FITC. Analysis with flow cytometry showed that approximately 90 % of the cells were labeled (Fig. 1). The hypothesis that the ArtinM CRD could mediate binding on spleen cells led us to perform a sugar inhibition assay. Based on the known selectivity of ArtinM sugar recognition, D-mannose was used as an ArtinM ligand, and D-galactose as a negative control. The proportion of ArtinM-labeled cells decreased in a dose-dependent manner when the lectin was preincubated with D-mannose, whereas D-galactose had no effect on ArtinM binding on spleen cells (see Fig. 1). The binding was inhibited up to 85–88 % when D-mannose concentrations varied from 20 to 50 mM, and was 50 % inhibited by 10 mM. Otherwise, 1 mM D-mannose or any D-galactose concentration tested had no significant effect on ArtinM binding to the spleen cell surface. These results suggest that glycans expressed on murine spleen cells are targeted by ArtinM.

### Mitochondrial activity in murine spleen cells stimulated by jArtinM and rArtinM

To investigate whether the ArtinM interactions with glycotargets on the spleen cell surface trigger cell activation, we assessed the mitochondrial activity of ArtinM-stimulated spleen cells by performing MTT assay. Two different ArtinM preparations were tested. The first was jArtinM, which is the native tetrameric protein purified from jackfruit seeds. The other was rArtinM, the recombinant counterpart protein obtained from lectin expression in *E. coli* BL21 and characterized as monomeric. At varying concentrations (0.1–625 nM), these preparations were used to stimulate spleen cell cultures for 12–72 h.

**Fig. 2:**
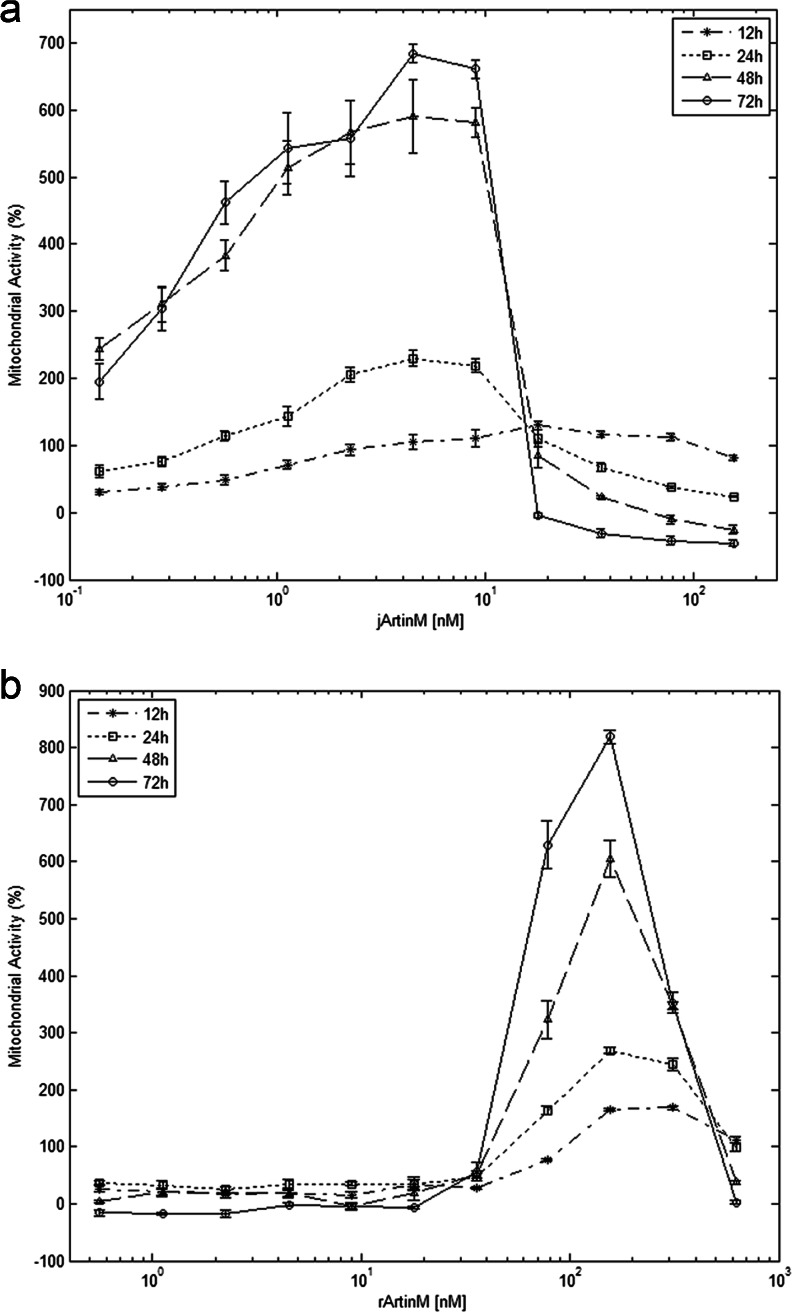
ArtinM stimulates mitochondrial activity of spleen cells in a dose-dependent manner. Murine spleen cells (1.5 × 10^6^ cells/mL) from BALB/c were distributed in 96-well microplates and incubated at 37 °C in a humidified atmosphere of 5 % CO_2_ and stimulated with jArtinM (**a**) or rArtinM (**b**) in concentrations of 0.14–156 or 0.56–625 nM, respectively. Non-stimulated spleen cells were used as negative controls. After 12, 24, 48, and 72 h of incubation, 3-(4,5-dimethyl-thiazol-2-yl)-2,5-diphenyltetrazolium bromide (MTT) was added to the culture medium; MTT reduction to insoluble purple formazan dye crystals was detected via absorbance reading at 570 nm. Mitochondrial activity was expressed as absorbance variation (in percentages) in relation to the negative control. Stimulation with Concanavalin A (49 nM) was used as a positive control, which provided the following absorbance variations: 114.9 ± 4.7 (12 h), 240.6 ± 40.58 (24 h), 852.7 ± 22.41 (48 h), and 704.6 ± 15.3 (72 h). The results represent 3 independent experiments and are expressed as means ± SEM

Increased mitochondrial activity of spleen cells was mostly observed after 48 and 72 h of stimulation. jArtinM augmented mitochondrial activity when used at concentrations of 0.14–9 nM, and maximum activity (closed to that provided by ConA, used as a positive control) was determined with 1.12–9 nM ArtinM (Fig. 2a). Stimulating similar mitochondrial activity required much higher concentrations of rArtinM. Maximum activity was determined with 156 nM rArtinM, which is a concentration 35 times higher than that of jArtinM required to induce the activity peak (Fig. 2b). No mitochondrial activity was detected when jArtinM concentrations were equal or superior to 18 nM, suggesting that high lectin concentrations may be toxic for the spleen cells (see Fig. 2).

### IL-2 production by spleen cells stimulated by jArtinM and rArtinM

**Fig. 3:**
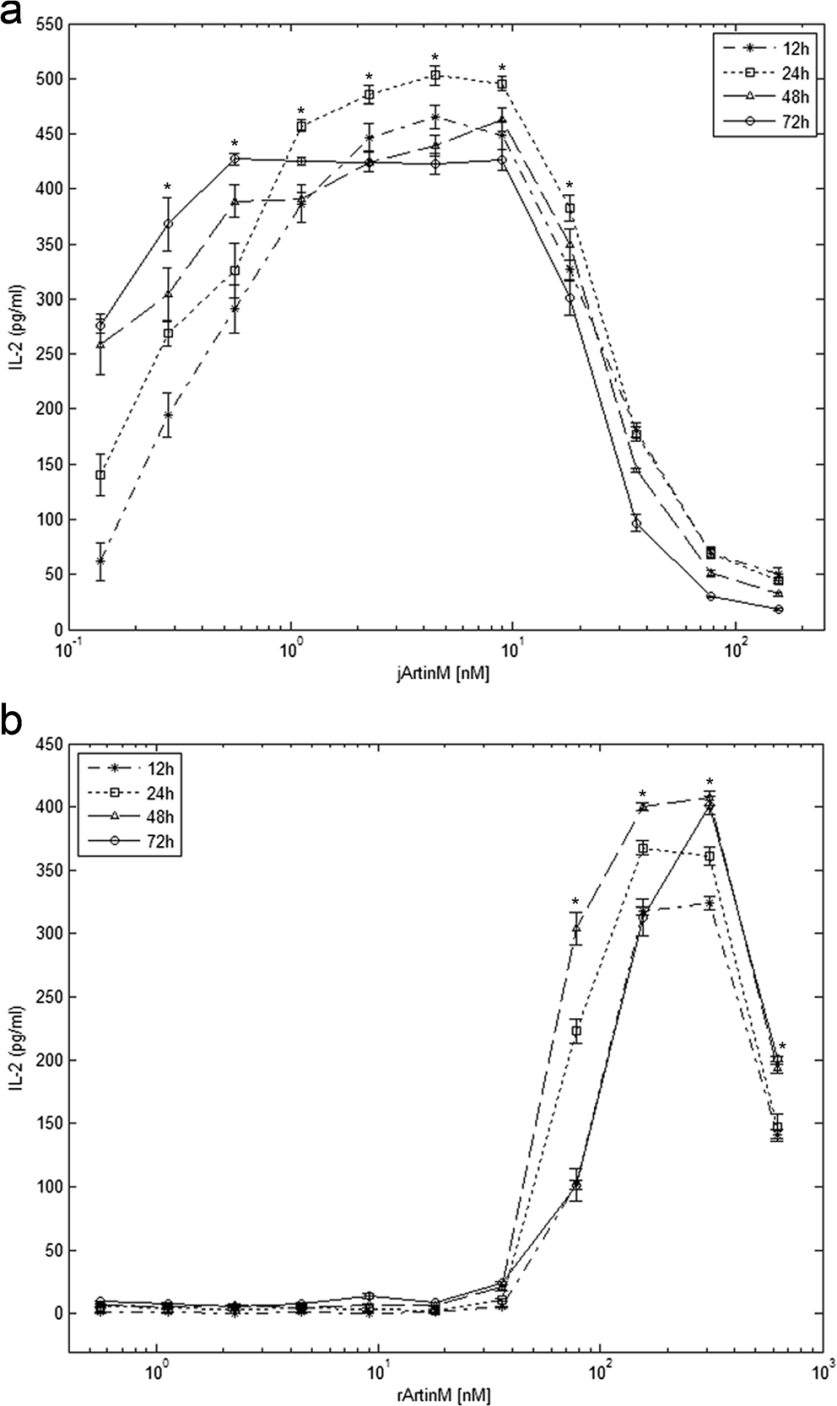
ArtinM stimulates interleukin-2 (*IL-2*) production by spleen cells in a dose-dependent manner. Spleen cells (1.5 × 10^6^ cells/mL) from BALB/c were distributed in 96-well microplates and incubated at 37 °C in a humidified atmosphere of 5 % CO_2_ and stimulated with jArtinM (**a**) or rArtinM (**b**) in concentrations of 0.14–156 or 0.56–625 nM, respectively. After 12, 24, 48, and 72 h of incubation, the cell culture supernatants were analyzed for IL-2 levels with enzyme-linked immunosorbent assay. Stimulation with Concanavalin A (49 nM), used as a positive control, provided the following IL-2 levels: 465.60 ± 4.49 (12 h), 495.8 ± 7.06 (24 h), 421.6 ± 4.37 (48 h), and 431.3 ± 6.73 (72 h). Non-stimulated spleen cells, used as negative controls (medium), provided the following IL-2 levels: 10.11 ± 2.04 (12 h), 10.24 ± 2.16 (24 h), 18.19 ± 3.29 (48 h), and 31.59 ± 4.49 (72 h). The results represent 3 independent experiments and are expressed as means ± SEM. *p < 0.05 compared to the medium for all incubation periods

Because ArtinM binds to glycotargets on murine spleen cells and increases mitochondrial activity, we investigated whether jArtinM or rArtinM stimulation induced IL-2 production. At concentrations from 0.14 to 36 nM, jArtinM significantly increased IL-2 production by murine spleen cells, yielding a bell-shaped dose–response curve that was more discernible when cells were stimulated for 24 h. Maximum IL-2 levels were determined with 2–9 nM jArtinM (Fig. 3a). Regarding the response to rArtinM, significant augmentation of IL-2 production was determined using 78–625 nM (Fig. 3b). Reaching peak IL-2 production required 4.5 nM jArtinM and 156 nM rArtinM. Therefore, a concentration of rArtinM 35 times higher than that of jArtinM was necessary to induce the highest levels of IL-2 production, which was close to that induced by 49 nM ConA (positive control).

### Spleen cell proliferation induced by jArtinM and rArtinM

**Fig. 4:**
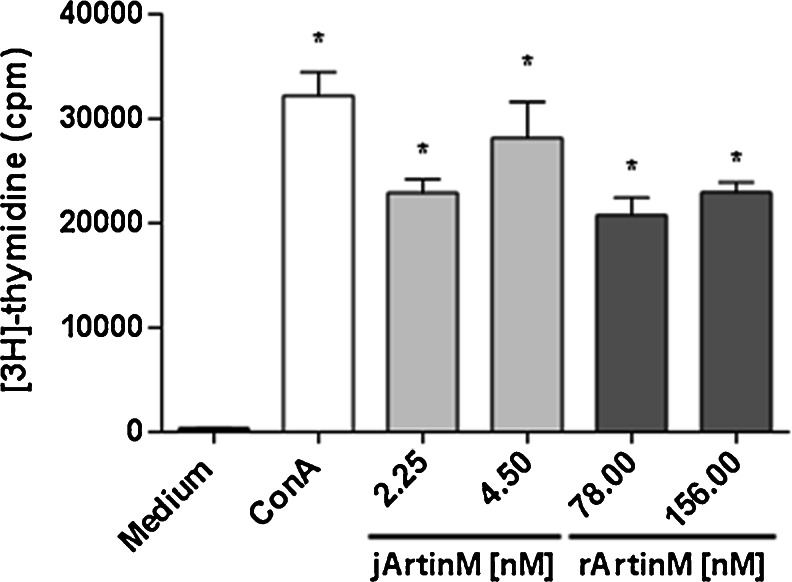
Spleen cell proliferation induced by ArtinM. Spleen cells (1.5 × 10^6^ cells/mL) from BALB/c were distributed in a 96-well microplate and incubated for 48 h at 37 °C in a humidified atmosphere with 5 % CO_2_ and stimulated with the indicated concentrations of jArtinM or rArtinM. Medium alone and Concanavalin A (*ConA*) (24.5 nM) were used as negative and positive controls, respectively. After 36 h of stimulation, [3H]-thymidine (0.5 μCi/well) was added and measured for incorporation (cpm) after 12 h of incubation. The results are expressed as means ± SEM. *Significant differences with *p* < 0.05 in relation to medium values

Because both jArtinM and rArtinM stimulate mitochondrial activity of murine spleen cells and IL-2 production, we assayed their capacity to induce cell proliferative response through the [3H]-TdR incorporation assay. The doses of jArtinM and rArtinM that induced maximal mitochondrial activity and IL-2 production were chosen as the lectin concentrations to be assayed for cell proliferation induction. We used 2.25 nM and 4.5 nM jArtinM and 78 nM and 156 nM rArtinM. The two doses of both lectin preparations promoted spleen cell proliferation in levels closed to those determined by the positive control, 24.5 nM ConA (Fig. 4).

### Carbohydrate recognition in jArtinM and rArtinM stimulation of spleen cells

**Fig. 5:**
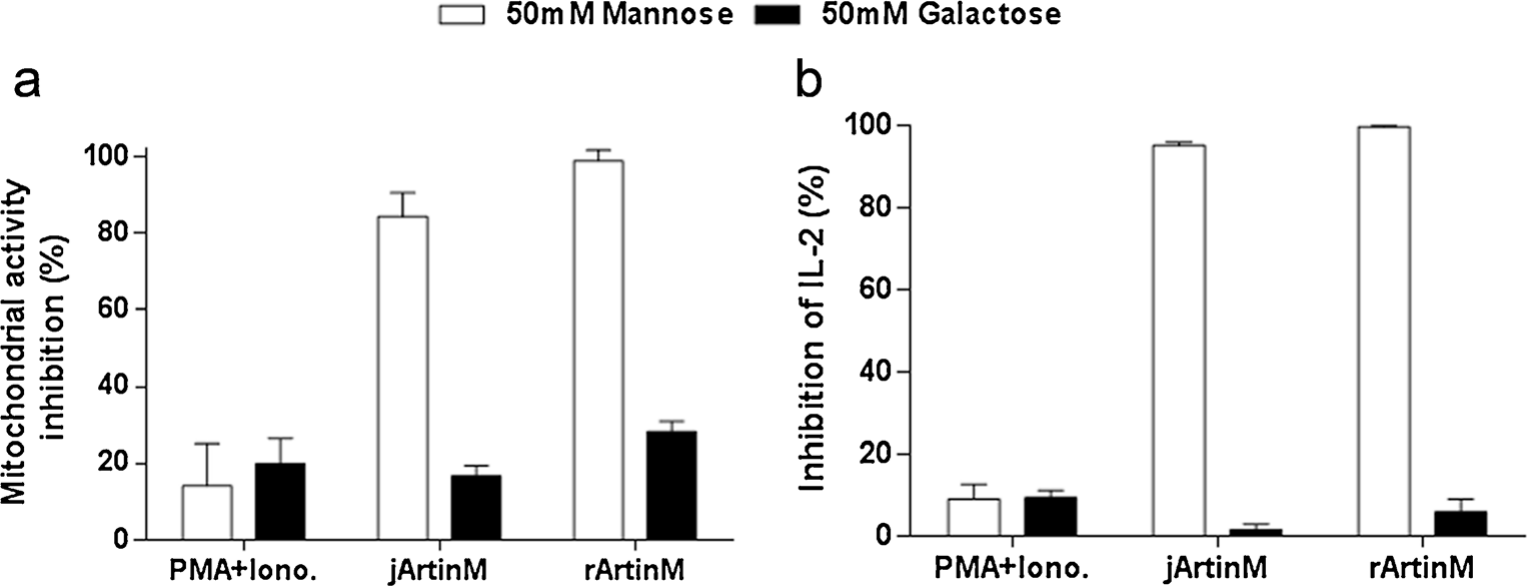
Spleen cell activation by ArtinM depends on carbohydrate recognition. Murine spleen cells (1.5 × 10^6^ cells/mL) obtained from BALB/c were distributed in a 96-well microplate and incubated at 37 °C in a humidified atmosphere of 5 % CO_2_. The cells were stimulated with jArtinM (2.25 nM) or rArtinM (78.00 nM) and were previously incubated or not with 50 mM D-mannose or D-galactose. Phorbol myristate acetate (*PMA*; 81 nM) plus ionomycin (1 μM) was used as a positive control stimulus. After 48 h of stimulation, mitochondrial activity (**a**) and IL-2 production (**b**) were determined. The results are expressed as % of inhibition, obtained by relating the measurements in the presence and absence of sugar for each stimulus. The results represent 3 independent experiments

Because ArtinM CRD accounts for binding on spleen cells, we evaluated the role of carbohydrate recognition in cell activation induction. The fact that mitochondrial activity and IL-2 production were stimulated by jArtinM and rArtinM made these parameters suitable for sugar inhibition assays. The pre-incubation of 2.25 nM jArtinM or 78.00 nM rArtinM with 50 mM D-mannose inhibited 80 % of the mitochondrial activity induced by both ArtinM preparations and blocked the lectin property of inducing IL-2 production by spleen cells. Otherwise, D-mannose did not modify the activities of the positive control, which was PMA plus ionomycin. We also observed that pre-incubation of spleen cells with 50 mM D-galactose had no effect on spleen cell activation induced by ArtinM. These results indicate that the jArtinM and rArtinM effects on spleen cells are mediated by sugar recognition (Fig. 5).

### Spleen cell activation induced by ArtinM does not depend on TLR2 or TLR4

**Fig. 6:**
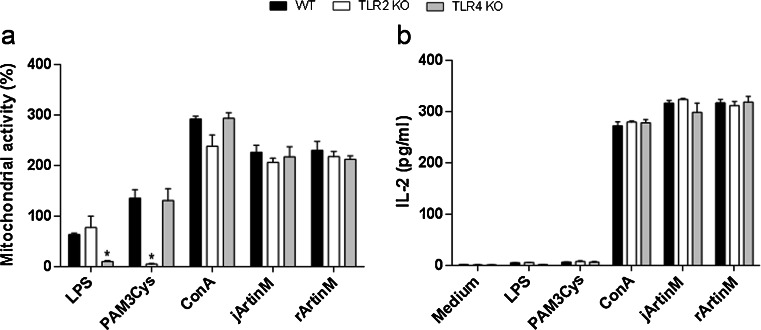
TLR2 or TLR4 does not mediate spleen cell activation by ArtinM. Murine spleen cells (1.5 × 10^6^ cells/mL) from C57BL/6 (*WT*), TLR2 KO, and TLR4 KO mice were plated onto 96-well microplates, incubated at 37 °C and 5 % CO_2_ in a humidified incubator, and stimulated for 24 h with 9 nM jArtinM or 312 nM rArtinM. Pam3Cys (TLR2 agonist, 1.5 μg/mL), LPS (TLR4 agonist, 1 μg/mL), or Concanavalin A (*ConA*) (49 nM), were used as positive controls for stimulation, whereas the medium alone was used as negative control. Next, the cells were analyzed for mitochondrial activity (MTT assay) (**a**) and IL-2 production (ELISA method) (**b**). Results are expressed as means ± SEM. *Differences in response from that of *WT* cells with *p* < 0.05 was considered as significant

Since ArtinM recognizes glycans on TLR2, we investigated whether this interaction could account for the murine spleen cell activation. For this, we evaluated ArtinM-mediated stimulation of spleen cells isolated from TLR2 KO mice. Spleen cells from TLR4 KO mice were also examined to exclude the possibility of LPS contamination in the ArtinM preparations. Typically, spleen cells were stimulated for 24 h with jArtinM (9 nM) or rArtinM (312 nM), and were analyzed for mitochondrial activity as well as IL-2 production. Both analyses showed that ArtinM as well as ConA similarly activated spleen cells from WT and KO mice (Fig. 6). Mitochondrial activity induced by LPS or Pam3Cys was absent in TLR4- or TLR2-deficient spleen cells, respectively. Since these agonists failed to induce IL-2 production after 24 h, we conclude that TLR agonists and lectins induced spleen cells activation through distinct mechanisms.

### Signaling molecules in jArtinM-and rArtinM-induced spleen cell activation

**Fig. 7:**
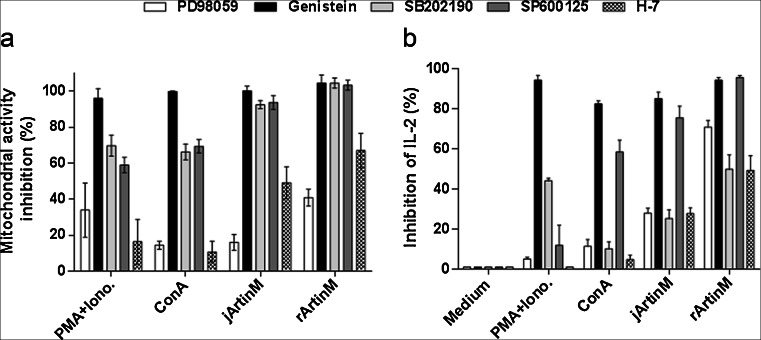
Effect of inhibitor agents of molecules accounting for cell signaling pathways on jArtinM- or rArtinM-induced activation of spleen cells. PD98059 [p42/44 mitogen-associated protein kinase (MAPK) inhibitor; 20 μM], genistein (protein tyrosine kinase inhibitor; 20 μg/mL), SB202190 (p38 MAPK inhibitor; 20 μM), SP600125 (c-Jun *N*-terminal kinase inhibitor; 25 μM), H-7 (protein kinase C inhibitor; 20 μM), or medium alone (absence of inhibitor) were used to pretreat, for 210 min, murine spleen cells (1.5 × 10^6^/mL) that were distributed in 96-well plates and maintained at 37 °C in a humidified atmosphere with 5 % CO_2_. After pretreatment, cells were stimulated with jArtinM (2.25 nM) or rArtinM (78 nM) for 48 h. PMA (81 nM) plus ionomycin (1 μM) or Concanavalin A (ConA) (49 nM) were positive controls for cell stimulation, whereas medium alone provided the negative control. The cells were analyzed for mitochondrial activity via MTT assay (**a**) and IL-2 production via ELISA (**b**). Measurements are expressed as means ± SEM, and the inhibition determined by each pharmacological agent was calculated by the ratio between the values obtained in the presence of a certain inhibitor and in its absence, represented by percentages

Considering that jArtinM and rArtinM have different oligomeric structures and induce spleen cell activation at distinct concentrations, we questioned whether they used the same or different signaling molecules to trigger the verified responses. Several pharmacological inhibitors (genistein, H-7, PD98059, SB202190, and SP600125) were then used to investigate the possible signaling molecules involved in responses to 2.25 nM jArtinM or 78 nM rArtinM. After 3 h 30 min of inhibitor treatment, cells were stimulated and analyzed for mitochondrial activity and IL-2 production. A similar profile of mitochondrial activity inhibition was associated with stimulation by jArtinM and rArtinM, showing that p38 MAPK, protein tyrosine kinase, and JNK are strongly involved in signaling triggered by both lectin preparations (Fig. 7a). Our results also demonstrated that IL-2 production was affected by protein tyrosine kinase inhibitor and JNK inhibitor (Fig. 7b) when stimulated by either jArtinM or rArtinM. Therefore, specific signaling molecules responsible for spleen cell activation were involved in the responses to both, native and recombinant ArtinM.

### ArtinM binding on murine T cells

**Fig. 8:**
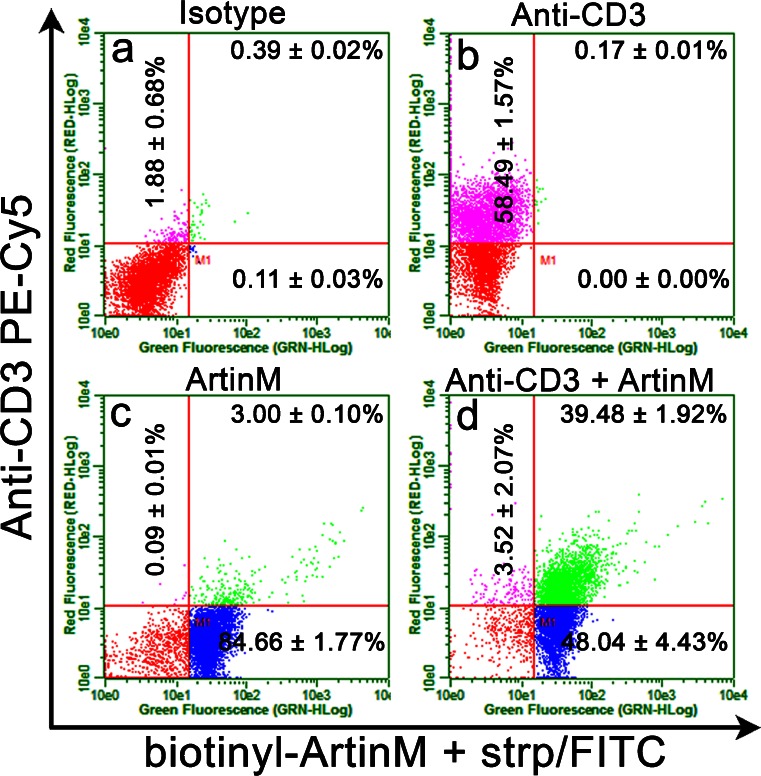
ArtinM binds to the surface of T lymphocytes. Spleen cells (1.5 × 10^6^/ml) were fixed and incubated for 40 min with anti-CD3 PE-Cy5 antibody (4 μg/mL) (**b**, **d**). After washing, the cells were incubated for 40 min with biotinylated ArtinM (15 μg/mL) (**c**, **d**). ArtinM binding was revealed by reaction for 40 min with streptavidin-FITC (5 μg/mL). Streptavidin-FITC and Isotype PE-Cy5 were used as a negative control (**a**). Cell fluorescence was analyzed with flow cytometry. Dot plots represent the percentage of positive cells obtained by biotinyl-ArtinM + strp/FITC or anti-CD3 PE-Cy5. The results are expressed as means ± SEM

Considering that spleen cells encompass distinct cell populations that include T lymphocytes, we investigated whether ArtinM recognized T cell glycans and induced the cell activation process observed when unseparated murine spleen cells were used. Through flow cytometry, we first analyzed jArtinM binding on spleen CD3^+^ cells. We verified that approximately 60 % of the spleen cell suspension was stained for CD3. Among these anti-CD3 stained cells, 97 % were targeted by jArtinM; i.e., they were double-labeled for CD3 and jArtinM binding (Fig. 8). A proportion of 88 % of the cell suspension was labeled with jArtinM, 48 % of them corresponding to double-positive cells. These data indicate that T lymphocytes are targeted by jArtinM and are candidates to undergo the activation process induced by the lectin.

### IL-2 production by CD4^+^ T cells stimulated by jArtinM and rArtinM

**Fig. 9:**
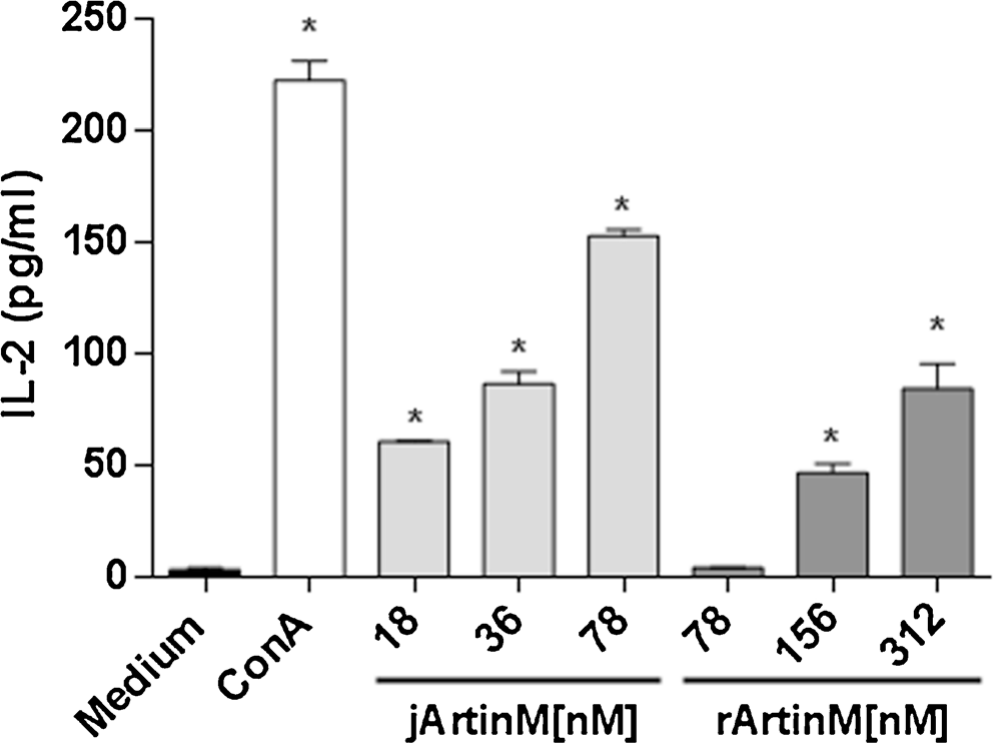
jArtinM and rArtinM enhances IL-2 secretion in CD4^+^ T cells. Purified CD4^+^ T cells (1 × 10^6^ cells/mL) from a spleen cell suspension of BALB/c mice were distributed in 96-well microplates and maintained at 37 °C in a humidified atmosphere with 5 % CO_2_. The CD4^+^ T cells were stimulated for 48 h with jArtinM (18, 36, or 78 nM) or rArtinM (78, 156, or 312 nM). Concanavalin A (49 nM) was used as a positive control of stimulation. Culture supernatants were analyzed for IL-2 production using ELISA, and the results are expressed as means ± SEM. *Significant differences when *p* < 0.05 compared with medium values

Because lymphocyte activation is manifested by IL-2 production, and CD4^+^ T lymphocytes constitute a major IL-2 cell source, we assayed the effect of jArtinM and rArtinM stimulation on purified CD4^+^ T cells. After 48 h of incubation with 18, 36, or 78 nM jArtinM or 78, 156, or 312 nM rArtinM, IL-2 production was induced in a dose-dependent manner. Figure 9 shows that similar levels of IL-2 were produced by CD4^+^ T cells stimulated with 36 nM jArtinM or 312 nM rArtinM, demonstrating that rArtinM is approximately ten times less effective than jArtinM for inducing lymphocyte activation.

### CD3 as a biologically relevant glycotarget of ArtinM

**Fig. 10:**
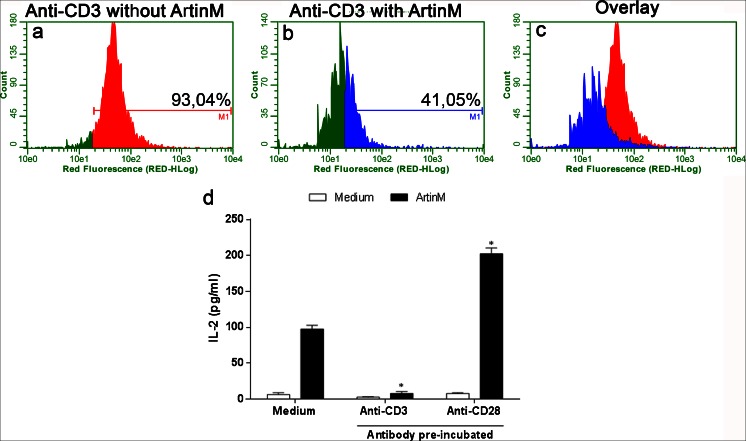
CD3 receptor as a glycotarget of ArtinM. (**a**–**c**) Purified CD4^+^ T cells (1.5 × 10^6^/mL) were fixed and incubated for 40 min with ArtinM (25 μg/mL). After washing, the cells were incubated for 40 min with an anti-CD3 PE-Cy5 antibody (10 μg/mL) and analyzed using flow cytometry. The histograms represent the percentage of cells positive for staining with the anti-CD3 PE-Cy5 antibody after preincubation with (**b**) or without (**a**) ArtinM. **d** Isolated CD4^+^ T cells (1 × 10^6^/mL) were incubated with the anti-CD3 antibody (5 μg/mL) or an anti-CD28 antibody (5 μg/mL) for 40 min and then stimulated with ArtinM (78 nM) for 48 h. IL-2 concentration in the culture supernatants was quantified by means of ELISA, and the results are expressed as mean ± SEM; *p < 0.05 compared to the cells that were preincubated with vehicle (culture medium) and then stimulated with ArtinM

The possibility that CD3 could be a glycotarget of ArtinM on CD4^+^ T cells was examined using an inhibition assay. Isolated CD4^+^ T cells, preincubated with or without ArtinM, were incubated with an anti-CD3 antibody. Flow cytometry analysis showed that ArtinM drastically inhibited the cell labeling with the anti-CD3 antibody (Fig. 10a–c). Because CD3 is known to be functionally important for T cell activation, we tested the ability of the anti-CD3 antibody to block the ArtinM-induced activation of CD4^+^ T cells. The cells were preincubated with or without the anti-CD3 antibody and then stimulated with ArtinM. An IL-2 assay in culture supernatants showed that the anti-CD3 antibody inhibited the stimulatory effect of ArtinM on CD4^+^ T cells (Fig. 10d) and pointed to the functional relevance of CD3 as a glycotarget of ArtinM. A specific antibody against CD28, a costimulatory molecule that also participates in the activation of T lymphocytes, was unable to block the activation of CD4^+^ T cells by ArtinM (Fig. 10d).

## Discussion

The known ArtinM property of stimulating Th1 immunity in vivo has been attributed to lectin interaction with TLR2 N-glycans on the surface of macrophages and dendritic cells. The present study is the first systemized effort to investigate the in vitro lectin effect on cells of adaptive immunity with the assumption that it could contribute to the immuno modulation determined by ArtinM administration. By stimulating murine spleen cells, ArtinM induces cell activation, as manifested by increased mitochondrial activity, IL-2 production, and proliferation. These activities were shown to depend on carbohydrate recognition and involve glycotargets on surface CD4^+^ T cells.

The lymphoproliferative properties of plant lectins have been reported extensively since the discovery that phytohemagglutinin (PHA) from *Phaseolus vulgaris* (Nowell 1960), pokeweed mitogen from *Phytolacca americana* (Farnes et al. 1964), and concanavalinA from *Canavalia ensiformis* (Wecksler et al. 1968) stimulate lymphocytes to undergo mitosis. The application of mitogenic lectins as tools in biomedical research gained force when PHA-activated human lymphocytes were shown to secrete a T cell growth factor, now known as IL-2. Subsequently, activated immune cells were shown to produce many other factors, now collectively denoted as cytokines.

It is well established that lymphocyte activation by lectins is initiated via binding to cell surface sugars, a fact verified here for ArtinM and reinforcing a previous observation by Benoist et al. (2009). We showed that the lectin binds on the surface of non-separated spleen cells as well as on isolated CD4^+^ T cells in a manner that depends on carbohydrate recognition, because D-mannose selectively inhibited ArtinM binding to the cell surface. Our preliminary results showed that ArtinM binds to Jurkat cells through carbohydrate recognition (data not shown), as has been demonstrated for Jacalin (Baba et al. 2007). Additional studies are underway to analyze the mitogenic and/or cytotoxic effects of jArtinM and rArtinM on human cells.

Clearly, binding to appropriately glycosylated receptors is insufficient to stimulate lymphocytes. Mitogenic lectins set in motion a signal transduction pathway that also functions on the antigen-dependent activation of lymphocytes. The key event in this pathway is protein tyrosine kinase activation, which mediates interactions with adapter proteins in a process that allows the enzymatic hydrolysis of phosphatidylinositol 4,5-biphosphate into diacylglycerol and inositol 1,4,5-triphosphate, both of which act as Ca^++^-dependent second messengers. Subsequently, the activation of two major pathways that involve Ras and protein kinase C results in the activation of MAPKs and extracellular signal-regulated kinases 1 and 2. The concerted action of the signal amplification triggers biochemical processes that result in the production and release of IL-2 (Smith-Garvin et al. 2009). Using inhibitors of signaling molecules, we showed that the increased mitochondrial activity and IL-2 production induced by ArtinM depend on the signaling molecules mentioned above. Additional experiments using purified T cells are necessary to further analyze and demonstrate the involvement of these molecules.

Mitogenicity of ArtinM on human PBMC were focused on the very first reports of ArtinM biological properties (de Miranda-Santos et al. 1991). Subsequent studies were primarily motivated by the discovery that ArtinM administration confers resistance against *L. major* infection in BALB/c mice (Panunto-Castelo et al. 2001). The studies demonstrated that ArtinM directly stimulates antigen-presenting cells to produce IL-12, providing an apparently full rational basis for ArtinM application as an efficient immunomodulatory agent. Although mandatory, evaluation of the ArtinM direct effect on cells of the adaptive immunity has been withdrawn. Indeed, correlation between the properties of several plant lectins of stimulating IL-12 production and inducing lymph proliferation has been examined previously and showed that only wheat germ agglutinin of *Triticum vulgare* induced IL-12 production without promoting cell proliferation or IL-2 release (Muraille et al. 1999). Here, we demonstrate that ArtinM-induced proliferation of murine spleen cells is independent of TLR2. Most of the lectins assayed by Muraille et al. (1999), and now verified for ArtinM, have been shown to stimulate both lymphocyte proliferation and IL-12 production, suggesting that they recognize specific oligosaccharides on the surface of the cells involved in adaptative and innate immunity.

The sugar-binding specificity of lectins is due to a limited segment of conserved amino acid residues that constitute their CRD (Drickamer 1988; Sharon and Lis 2004). An appropriate CRD structure is required from recombinant lectins to reproduce the sugar specificity of their native counterparts. This requirement was satisfied by rArtinM, as verified by glycoarray studies (Pranchevicius et al. 2012) and reinforced by the similar kinetic rates and affinity equilibrium constants provided by the interaction of jArtinM and rArtinM with *N*-glycans of horseradish peroxidase glycoprotein (Pesquero et al. 2010). However, they differ in terms of avidity owing to diversity in their tertiary structures: jArtinM is organized as a tetramer, whereas rArtinM is monomeric (Pranchevicius et al. 2012). Nonetheless, these features are insufficient to explain the distinct concentrations required of jArtinM and rArtinM to activate spleen cells. The quotients between the concentrations required to induce mitochondrial activity, IL-2 production, and cell proliferation are much higher (>30) than that between the number of CRDs per jArtinM and rArtinM molecule (=4). Regardless, these preparations stimulated the same responses on spleen cells, and in vivo administration of rArtinM was as effective as jArtinM in conferring protection against *P. brasiliensis* (Coltri et al. 2008). We hypothesized that ArtinM tetramer, as a multivalent ligand, has superior efficiency owing to a greater capability to cluster receptors on the cell surface in a process that optimizes the triggered cell signaling, as reported for T cell receptor (TCR) containing microclusters that generate and sustain T-cell activation (Yokosuka et al. 2005).

To characterize the mechanisms of immunomodulation induced by external agents, we must investigate the effect they exert on immune cell populations. The various T CD4^+^ effector/regulatory subpopulations deserve special attention. *Naive* CD4^+^ T cells may differentiate into any of several lineages of Th cells (Th1, Th2, and Th17) or into induced regulatory T cells. Th1 cells make interferon-γ as a signature cytokine and are also IL-2 producers; in addition, IL-12, mostly produced by antigen-presenting and natural killer cells, plays a central role in Th1 differentiation (Zhu and Paul 2010). In the case of ArtinM induced-Th1 immunity, the stimulus of IL-12 production has been thoroughly examined. The present study suggests that the Th1 immunity (known to be induced in vivo by ArtinM) involves ArtinM’s direct effects on differentiation of naive CD4^+^ T cells into the Th1 lineage. This is because lectin binds to CD4^+^ T cells and induces IL-2 production. This idea is reinforced by the observation that jArtinM induces IFN-γ production by CD4^+^ T cells (data not shown). Moreover, the mechanism of CD4^+^ T-cell activation by ArtinM involves interaction with CD3, which was shown to be a functionally relevant glycotarget of ArtinM in the process of activation of the lymphocytes. Although our results do not rule out participation of other ArtinM glycotargets in driving the activation of CD4^+^ T cells, we can state that CD3 is an essential participant because its blockage with a specific antibody inhibited the response to ArtinM by more than 90 %.

In summary, our study shows that ArtinM binding to murine spleen cells is followed by increased proliferation, mitochondrial activity, and IL-2 production in a carbohydrate recognition-dependent manner. CD4^+^ T cells are clearly targeted by the lectin, providing a direct mechanism of inducing Th1 immunity. It is added to the indirect mechanism provided by ArtinM targeting of antigen-presenting cells, which accounts for increased IL-12 production and stimulation of Th1 differentiation. This double Th1 stimulation may decisively contribute to the efficiency of the in vivo immunomodulation induced by ArtinM administration. The present work opens new perspectives in understanding the full mechanism of immunomodulation induced by carbohydrate recognition.
